# Functional aspects of the brain lymphatic drainage system in aging and neurodegenerative diseases

**DOI:** 10.7555/JBR.37.20230264

**Published:** 2024-03-02

**Authors:** Yan Chen, Xiaoxin He, Jiachen Cai, Qian Li

**Affiliations:** 1 Jiangsu Key Laboratory of Neurodegeneration, Nanjing Medical University, Nanjing, Jiangsu 211166, China; 2 Shandong Institute of Brain Science and Brain-inspired Research, Shandong First Medical University & Shandong Academy of Medical Sciences, Jinan, Shandong 250117, China; 3 Center for Global Health, Nanjing Medical University, Nanjing, Jiangsu 211166, China

**Keywords:** glymphatic system, meningeal lymphatic vessels, aging, neurodegenerative diseases, non-pharmacological therapies

## Abstract

The phenomenon of an aging population is advancing at a precipitous rate. Alzheimer's disease (AD) and Parkinson's disease (PD) are two of the most common age-associated neurodegenerative diseases, both of which are primarily characterized by the accumulation of toxic proteins and the progressive demise of neuronal structures. Recent discoveries about the brain lymphatic drainage system have precipitated a growing body of investigations substantiating its novel roles, including the clearance of macromolecular waste and the trafficking of immune cells. Notably, aquaporin 4-mediated glymphatic transport, crucial for maintaining neural homeostasis, becomes disrupted during the aging process and is further compromised in the pathogenesis of AD and PD. Functional meningeal lymphatic vessels, which facilitate the drainage of cerebrospinal fluid into the deep cervical lymph nodes, are integral in bridging the central nervous system with peripheral immune responses. Dysfunction in these meningeal lymphatic vessels exacerbates pathological trajectory of the age-related neurodegenerative disease. This review explores modulatory influence of the glymphatic system and meningeal lymphatic vessels on the aging brain and its associated neurodegenerative disorders. It also encapsulates the insights of potential mechanisms and prospects of the targeted non-pharmacological interventions.

## Introduction

The elucidation of the glymphatic system represents a seminal development in neurobiology when it was first described in murine models by Professor Maiken Nedergaard's team in 2012^[1]^. This intricate system facilitates the ingress of cerebrospinal fluid (CSF) into the brain parenchyma *via* perivascular space (PVS) surrounding penetrating arteries. Subsequently, it orchestrates the collection and removal of intracerebral metabolites through similar outflow tracts. A critical component of this system is the presence of aquaporin 4 (AQP4) on the astrocytic endfeet, which underpins the exchange flow between CSF and interstitial fluid (ISF). In an advancing stride in 2015, Professor Jonathan Kipnis's group^[2]^, unveiled the existence of functional meningeal lymphatic vessels lining the dural sinuses of mice, bearing molecular signatures akin to peripheral lymphatic endothelial cells. These meningeal conduits are charged with the conveyance of metabolites and immune cells from the CSF to the peripheral deep cervical lymph nodes (dCLNs). Their dysfunction has been posited as a potential culprit in an array of conditions, including multiple sclerosis^[3]^, Alzheimer's disease (AD)^[4]^, and neurological disorders concomitant with primary lymphedema^[5]^.

In a recent study in January 2023, Professor Nedergaard's team^[6]^ further refined our understanding of the brain lymphatic drainage system. They identified a previously unrecognized fourth meningeal layer, termed the subarachnoid lymphatic-like membrane (SLYM), which was distinct from the known pachymeninx, arachnoid and pia, and was reminiscent of the mesothelial membranes encasing adjacent organs. Notably, the SLYM bifurcated the subarachnoid space and housed a diverse array of immune cells, serving a functional role intimately linked to the venous sinus endothelium, thus facilitating material transfer between the CSF and venous blood^[6]^. The discovery of the dual role of SLYM as an immune barrier and conducted for fluid transport may provide some novel aspects of central nervous system (CNS) pathologies.

Since the function of SLYM still needs to be verified in physiological and pathological situations, the glymphatic system and meningeal lymphatic vessels have been extensively studied. This review seeks to delineate the divergences in anatomical configuration and fundamental morphology of brain lymphatic vessels across typical neurological model organisms and humans. Such comparative insights are indispensable for deepening our understanding of the brain lymphatic drainage apparatus and its overarching influence on neurological health.

## Anatomical and physiological diversity of the brain lymphatic drainage system

### Rodents

**Figure 1:**
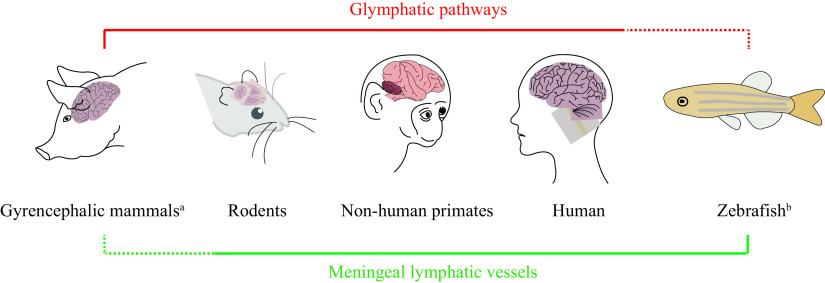
The conservatism of the brain lymphatic system among species in the current study.

The existence of the glymphatic system is a well-established fact across various species, extending beyond rodents^[1,7–8]^, which include model organisms such as pigs^[9]^, zebrafish^[10]^ and the human brain^[11–13]^, playing a critical role in the elimination of cerebral metabolites. Nevertheless, comprehensive reports delineating the intricate anatomical and functional distinctions of this system across different species remain sparse (***Fig. 1***). Advancements in the live tracing methodologies and imaging technologies have been pivotal. In a study by He *et al* in 2022^[14]^, a meticulous portrayal of the entire glymphatic architecture was achieved, alongside a three-dimensional reconstruction of the CSF flow pathways throughout the mouse brain, utilizing fluorescence micro-optical sectioning tomography. Following the intracisternal injection of a fluorescent tracer, discernible variations in glymphatic circulation across brain regions were observed. Priority distribution of tracer indicated a predominance of the CSF influx on the brain parenchymal ventral aspect, especially in those regions such as the medulla oblongata, pons, hypothalamus, olfactory bulb, and ventral cortex, with many of these areas being contiguous to the circle of Willis^[14]^. Such observations underscore the region-specific nature of the glymphatic flow, demonstrating a preferential perfusion with areas hosting sizeable vertical penetrating arteries or close to the subarachnoid space. Such a detailed resolution of the glymphatic system offers invaluable insights into potential subtle anomalies that may arise from neurological diseases.

In parallel, the detailed morphology of meningeal lymphatic vessels in mice has been comprehensively characterized in another study^[15]^. From an anatomical standpoint, these vessels are categorized into two primary subsets: the dorsal and the basal lymphatics. The basal meningeal lymphatics, running alongside the sigmoid sinus (SS), are characteristically broader, featuring an extensive network of capillary branches and blunt termini adorned with oak leaf-shaped lymphatic endothelial cells and interspersed lymphatic valves^[15]^. Further functional examination through the dynamic contrast-enhanced magnetic resonance imaging (MRI) of the CSF has ascertained that the structural composition of the basal lymphatics inherently facilitates more efficient absorption and elimination of the CSF, compared with their dorsal counterparts^[16]^.

### Gyrencephalic mammals

In the realm of experimental neurological research, gyrencephalic mammals such as pigs, dogs, and rabbits are frequently employed because of their highly gyrified brains. Studies have provided evidence of a protective role of the glymphatic system in porcine subjects. CSF tracers, when introduced, permeate along the cerebral sulci and gyri. The folding architecture of the porcine brain promotes CSF diffusion and facilitates a quadrupling of PVS flow density compared with mice^[9]^. Notably, glymphatic drainage is the most active during sleep, with CSF influx corresponding with the systolic phase of the cardiac cycle^[17]^. With humans averaging 33.3% of a 24-hour cycle asleep, pigs of 32.6%, rhesus monkeys of 49.2%, and mice of 44% with more scattered sleep patterns, the glymphatic system in pigs serves as an appropriate intermediary for translational studies to humans^[18]^. Thus, the glymphatic system in pigs was a suitable intermediate species between mice and humans, and the understanding of the glymphatic system may translate to the human system.

Furthermore, in models of cerebral ischemia spanning multiple gyri, there is a marked reduction in glymphatic drainage within boundary regions of the middle cerebral and anterior arteries, which are areas vulnerable to cerebral ischemic stroke, bolstering the rationale for targeting glymphatic drainage in stroke therapy^[19]^.

### Zebrafish

The zebrafish, transparent in tissue and rapid in reproductive cycling, provides an excellent model for vascular and lymphatic studies. In the groundbreaking work by scientists Neil I Bower^[20]^, Max van Lessen^[21]^, and Marina Venero Galanternik *et al*^[22]^ characterized lymphatic vessels in this model in 2017, they found that the meningeal mural lymphatic endothelial cells (muLECs) expressed markers akin to peripheral lymphatic endothelial cells and were crucial to the meningeal vascular system of a zebrafish. Sprouting from the midbrain's endothelial rings during larval stages, normal development of these meningeal mural lymphatic endothelial cells relies on a vascular endothelial growth factor C (VEGFC)-vascular endothelial growth factor D (VEGFD)-collagen and calcium-binding EGF domain-containing protein 1 (CCBE1)-vascular endothelial growth factor receptor 3 (VEGFR3) signaling pathway. Studies have also implicated these cells in angiogenesis within the meninges, laying a foundation for future research into lymphovascular-vascular transdifferentiation^[20]^.

Professor Luo's team in 2019^[23]^ observed that after cerebral vascular injury, lymphatic endothelial cells invaded the damaged brain parenchyma, aiding in ISF drainage and potentially providing a scaffold for new vascular growth, with apoptosis of these vessels occurring post-regeneration. Furthermore, it is found that the earliest emergency neovascularization formation was transdifferentiated from endogenous meningeal lymphatics in the damaged brain region after cerebral infarction. This process was dependent on Notch signaling activation and further regulated by the inhibitory EphB4a/EphrinB2a^[24]^. This study provided new clues for post-ischemic drug development, especially the promotion of meningeal lymphatic vessel transdifferentiation into cerebral vessels *via* the intervention of the EphrinB2a/EphB4a/Notch signaling pathway, which may be a significant clinical prospect.

### Non-human primates and humans

Similar to the findings in other species, the distribution of AQP4 in the human brain, as observed in the autopsy samples, is affected by age, with alterations correlated with cognitive function^[25–26]^. The regional localization of AQP4 in the prefrontal cortex of the elderly is significantly altered, as evidenced by a distinct pattern of laminar heterogeneity in the AQP4 expression in the top cerebral gyrus layer and the deep cerebral sulcus. In the top gyrus layer of the elderly, the AQP4 expression is absent between layers Ⅱ and Ⅳ of the cortices, whereas the AQP4 immunopositivity is shown between layers Ⅴ and Ⅵ of the gray matter-white matter borders. Deep in the cerebral sulcus of the elderly, a strong immunopositivity for AQP4 is shown in the cortical layer Ⅱ, while the AQP4 expression is reduced in deeper sites. Moreover, the aforementioned altered AQP4 localization is correlated with cognitive function^[27]^.

The presence of meningeal lymphatics in humans and non-human primates (common velvet monkeys) has also been identified by using the high-resolution MRI. These lymphatic vessels along the dural venous sinus are similar to those found in rodents. Moreover, the meningeal lymphatics showed typical positive lymphatic endothelial cell markers in primates by immunohistochemistry^[28]^.

Collectively, these insights underscore the significance of species-specific anatomical and functional distinctions of the lymphatic system in the brain, highlighting the connection between its dysfunctions and the pathophysiology of aging and neurodegenerative conditions^[15]^.

## The function of the brain lymphatic system in aging and age-related neurodegenerative diseases

Continuing this trajectory, we further delve into the roles and interconnections between glymphatic pathways and meningeal lymphatics in the context of aging and prevalent age-associated neurodegenerative diseases, which will stand to inform novel therapeutic approaches targeted at these critical systems.

### Natural aging

Aging represents a customary physiological occurrence. With the progression of age, the microglia's capacity to phagocytose cellular remnants, lipid droplets, and β-amyloid (Aβ) diminishes^[29–31]^, culminating in an extensive accumulation of toxic proteins within the brain parenchyma^[32]^. The efficacy of glymphatic transport was appraised by introducing radioactive markers into the cisterna magna of wild-type mice of varying months of age in a study, in which tracer penetration within the brains of middle-aged (10–12 months) and elderly mice (18–20 months) was found significantly inferior, compared with their youthful counterparts (2–3 months), and the elderly mice displayed the most marked decrease, underscoring a deterioration in glymphatic drainage concurrent with aging^[33]^.

Mounting evidence has indicated that a decline in the meningeal lymphatic vessel coverage and drainage function hastens cognitive decline in the aged mice^[34–35]^. Furthermore, the aged mice were found to display no significant morphological alterations in the capillary lymphatic vessels of ear skin, trachea and diaphragm, when compared with young mice, with only minor modifications observed in the lymphatic valves of skin-collecting lymphatic vessels, suggesting that the age-related changes in lymphatic vessels are organ-specific^[15]^. The lymphatic valve, which is pivotal in regulating the maturation of collecting lymphatic vessels and the flux of lymphatic fluid^[36–38]^, was observed aberrant in its distribution of type Ⅳ collagen and diminished in number within the basal lymphatic vessels of older mice^[15]^. Notably, lymphatic vessel valves in younger mice were reported elongated and densely aggregated, yet in older mice, they were more dispersed^[15,36]^. These observations infer a reduction in the lymphatic flow at the basal lymphatic vessel level in aged mice, indicating that ameliorating meningeal lymphatic drainage facilitates age-related pathophysiological processes.

### Alzheimer's disease (AD)

AD, the predominant form of the age-associated dementia, accounts for 60%–80% of all dementia instances^[39]^. So far, the immunotherapy targeting Aβ has demonstrated a limited efficacy. Aducanumab, in particular, has been shown to diminish the Aβ load in both AD model mice and mild AD patients^[40]^, which received the U.S. Food and Drug Administration approval for clinical use in 2021^[41]^. However, the efficacy of aducanumab in enhancing the clearance of parenchymal Aβ was attenuated when meningeal lymphatic drainage was impaired, as evidenced in a 5 × FAD mouse model; conversely, the treatment with VEGFC augmented the effect of monoclonal antibodies on the Aβ clearance and alleviated cognitive deficits of 5 × FAD mice^[42]^. These indicate that a combined approach of the enhanced meningeal lymphatic drainage and anti-Aβ antibodies may yield the improved clinical outcomes in the AD treatment.

AQP4 facilitates the CSF-ISF exchange and plays a pivotal role in the clearance of neurotoxic proteins, such as Aβ^[1]^. The reduction in AQP4 expression was found to exacerbate the accumulation of brain Aβ and cognitive deficits in the APPswe/PS1dE9 (APP/PS1) mice^[43]^; moreover, the loss of perivascular AQP4 localization might lead to the pathological process of AD in the human population. It was also confirmed that cortical perivascular endfoot mislocalization of AQP4 was correlated with the Aβ deposition, rather than the localization of AQP4 limited to non-perivascular fine processes of astrocytes that damaged the CSF-ISF exchange and the Aβ clearance^[44]^.

### Parkinson's disease (PD)

PD is the second most prevalent neurodegenerative condition, affecting approximately 2%–3% of individuals over 65 years of age^[45]^. In A53T mice of the PD model, the accumulation of perivascular α-synuclein (α-Syn) and compromised AQP4 polarization were observed in the substantia nigra; moreover, the disruption of brain lymphatic drainage exacerbated α-Syn accumulation, glial activation, dopaminergic neuron loss, and motor impairments in these mice^[46]^. In addition, by using the dynamic contrast-enhanced MRI, studies have shown an impaired meningeal lymphatic drainage in idiopathic PD patients, compared with patients with atypical Parkinsonian syndrome, suggesting that therapeutic enhancement of this drainage may mitigate the progression of PD^[47]^.

Sleep disturbances, including insomnia, atypical rapid eye movement sleep behavior, central sleep apnea syndrome and restless legs syndrome, are common non-motor symptoms in PD patients^[48–49]^. Mouse studies indicate a negative correlation between electroencephalogram delta wave activity and glymphatic clearance^[50]^. Patients with PD often exhibit a heightened delta wave activity during sleep^[51–52]^. These suggest a critical role of the glymphatic system in PD-related sleep disorders. Moreover, the progression of α-Syn during PD's early motor symptomatic phase may be confined to the midbrain and limbic areas. Therefore, enhancing sleep-related interventions targeting glymphatic clearance of α-Syn may be beneficial for PD progression.

## Potential mechanisms regulating the function of the brain lymphatic system

### Glymphatic system

**Figure 2:**
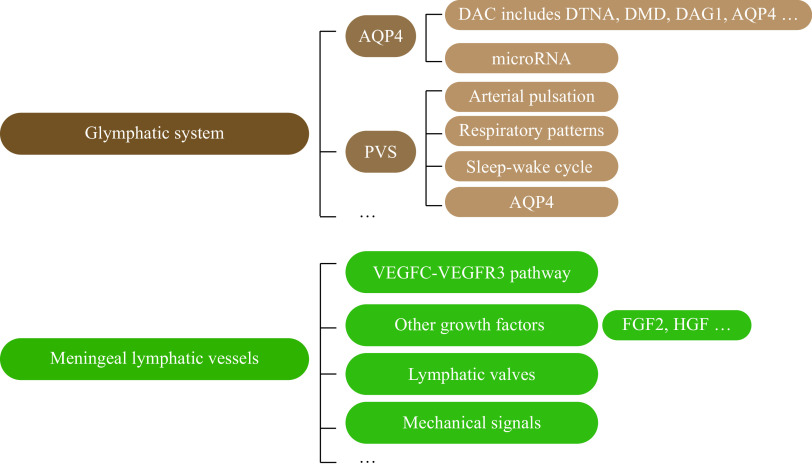
Potential mechanisms regulating the function of the glymphatic system and meningeal lymphatic vessels.

The glymphatic system facilitates the movement of CSF into the brain *via* the periarterial space, gathering metabolites from brain parenchyma, and then expels ISF through the perivenous space. Historical accounts by Virchow-Robin and Robin CP revealed the presence of fluid-filled tubular structures that surround the parenchymal capillaries, penetrating arteries and veins, which are now known as the Virchow-Robin space or PVS^[53–54]^. These spaces permit a rapid exchange between ISF and CSF without transgressing the endothelial cell layer, which is stringently regulated and is reliant on the expression of AQP4^[55]^. With these anatomical features, it became possible to regulate glymphatic drainage through AQP4 and PVS (***Fig. 2***).

#### AQP4

AQP4 predominantly exists in two isoforms, M1 and M23. Although they share comparable water transport abilities, their propensities for aggregation and their distribution on astrocytic membranes vary in the mouse brain parenchyma^[56–57]^. The presence of AQP4 at the astrocytic end-feet encasing blood vessels is contingent upon the dystrophin-associated protein complex (DAC). Principal constituents of this complex, including genes such as *DTNA*, *DMD*, *DAG1*, and *AQP4*, show high expression levels in astrocytes^[58–59]^. Intriguingly, the transcript levels of *Dag*, *Dtna*, and *Aqp4* were observed to be increased during daylight hours, compared with nighttime in mice; moreover, an upsurge in the expression of critical DAC components, including AQP4, correlated with a heightened glymphatic influx throughout the day; although diurnal variations did not affect the levels of M1 or M23 type AQP4, there was a tendency for the ratio of M1 to M23 to decline during the day^[60]^. These findings suggest that perivascular polarization of AQP4 supports the circadian rhythm of CSF distribution. Additionally, microRNA-130a was found to repress the level of AQP4-M1 to restore AQP4 polarity against AD, which indicates a novel potential treatment for AD^[61]^.

#### PVS

The PVS was widely considered to be limited by the pial surface border and extended down the vasculature to the capillaries, forming a conduit for the efflux of metabolic by-products and the interchange of CSF and ISF within the parenchyma^[62]^. However, the high-resolution 7T MRI scans have uncovered regional anatomical variations in the PVS within the human brain. Notably, the PVS within the basal ganglia appears to have a direct connection to the basal subarachnoid space, whereas, in the centrum semiovale, the PVS follows the vessels emanating from the dorsal cortex deep into the brain^[63]^. Deep perforating vessels from the base of the brain form collateral anastomoses with cortical and medullary vessels on the dorsal side in the so-called watershed zones, which play an important role in small vascular disease (SVD) and other pathological conditions. As reported, the SVD burden was associated with a poor recruitment of collaterals in large-vessel occlusive stroke, thus patients with poor collaterals were more likely to have an enlarged PVS and a higher SVD burden score^[64]^.

Studies indicate that the PVS is pivotal in CSF circulation and drainage, with the flow within the PVS being influenced by factors such as arterial pulsation^[65]^, respiratory patterns^[66]^, sleep-wake cycle^[17]^, and AQP4^[55]^. The rigid encasement provided by the skull facilitates the transmission of arterial pressure waves throughout the brain, resulting in discernible pulsatile fluid movement from the microvasculature to venous outflows. These pulsations are instrumental in purging the brain parenchyma of fluids and metabolic waste^[67]^. The influence of vascular pulsation on PVS drainage was demonstrated through the *in vivo* two-photon imaging in mice, illustrating how tracers injected into the cisterna magna migrated along the PVS^[68]^. Additionally, inspiration was shown to augment CSF influx into the ventricles, whereas expiration had the reverse effect^[66]^. This CSF movement was found most evident in the occipital foramen magnum and the spinal epidural vein^[69]^. Human subjects administered with gadolinium into the lumbar CSF exhibited the increased nocturnal PVS gadolinium concentrations, despite a disrupted sleep due to the repeated MRI procedures^[11]^, indicating that the sleep-wake cycle exerts an influence on PVS clearance functions. In contrast, while a burgeoning evidence points to that AQP4 enhances the entry of CSF from the PVS into the ISF^[55]^, the efficacy of AQP4 in promoting the ISF clearance remains a subject of debate^[70]^.

### Meningeal lymphatic vessels

Meningeal lymphatics have been reported to transport immune cells and remove metabolic waste as an efficient pathway in the CNS, which may be potential therapeutic targets for neurological disorders^[2,34,55,71]^. The plasticity and regenerative potential of meningeal lymphatic vessels promise new perspectives for the modulation of brain lymphatic drainage and the precise treatment of CNS pathologies^[72]^.

#### VEGFC-VEGFR3

Previous studies reported that the *K14-VEGFR3-Ig* transgenic mice, exhibiting an impaired VEGFC/D-VEGFR3 signaling, displayed hypoplastic meningeal lymphatic vessels; additionally, embryos lacking the *Vegfc* allele succumbed before meningeal lymphatic vessel development, indicating that *Vegfc* plays a pivotal role in meningeal lymphatic development^[55,73]^. It has been reported that normal meningeal lymphatic development requires at least 50%–75% of *Vegfc* mRNA levels in the wild-type mice; consequently, meningeal lymphatic vessels, compared with the skin or intestinal lymphatic vessels, are more sensitive to the decreased VEGFC levels^[73–74]^. Treatment of human lymphatic endothelial cells with recombinant human VEGFC protein results in a significant increase in tube formation^[75]^. VEGFR3 functions as the receptor for VEGFC, and subsequent investigations have revealed that obstructing the VEGFC-VEGFR3 pathway hinders the development of meningeal lymphatics and the formation of meningeal lymphatic vessels in adult mice^[76]^. Sunitinib was found to suppress VEGFR3 tyrosine kinase activity *in vivo*, without affecting pre-existing lymphatic vessels^[74]^. A two-week course of sunitinib treatment resulted in a reduction of paranasal lymphatics, particularly in the superior sagittal sinus region; however, some lymphatic vessels showed a renewed growth after four weeks off the drug^[72]^. These findings suggest a plasticity of meningeal lymphatic vessels.

#### Other growth factors

Although VEGFC is indispensable for regulating lymphangiogenesis, it is important to note that several other growth factors also induce lymphangiogenesis. For instance, in zebrafish, VEGFD has been identified as essential for facial lymphangiogenesis^[77]^. Ectopic expression of several other growth factors, such as fibroblast growth factor 2 (FGF2)^[78]^, vascular endothelial growth factor^[79]^, platelet-derived growth factor^[80]^, epidermal growth factor^[81]^, insulin-like growth factor-1^[82]^ and hepatocyte growth factor (HGF)^[83]^, also induce lymphangiogenesis in mouse tissues. For example, blocking VEGFC/D signaling inhibits lymphangiogenesis induced by FGF2^[78]^, angiopoietin 1^[84]^, and HGF^[85]^, indicating that these growth factors induce lymphangiogenesis by operating through the VEGFC/D pathway. In contrast, the ectopic expression of platelet-responsive protein-1^[86]^, pigment epithelium-derived factor^[87]^, and transforming growth factor^[88]^ has been found to inhibit lymphangiogenesis in mouse tissues. Moreover, in addition to direct effects on lymphatic endothelial cells, some growth factors may also act by recruiting leukocytes to produce VEGFC/D^[89]^. Furthermore, it is conceivable that growth factors induce VEGFC expression in vascular endothelial cells or associated smooth muscle cells, leading to lymphangiogenesis through a paracrine pathway^[78,85]^.

#### Lymphatic valves

The basal meningeal lymphatics are important "hotspots" for the drainage of macromolecules, similar to the collecting lymphatics in the peripheral system, containing lymphatic valves that are essential for the unidirectional flow of lymphatic fluid^[15]^. Dysfunction of these lymphatic valves leads to abnormal lymphatic vessel morphology and, subsequently, lymphedema^[37,90]^. Furthermore, Prospero homeobox 1 (PROX1), forkhead box C2 (FOXC2), and GATA binding protein 2 (GATA2) are vital transcription factors responsible for the maintenance of lymphatic vessel valves^[36,38,91]^. The expression levels of both PROX1 and FOXC2 were observed to be significantly decreased in the meningeal lymphatic vessels of aged mice; additionally, the level of PROX1 in lymphatic endothelial cells decreased with age^[15]^, suggesting that the functional impairment of the basal lymphatic vessels is further compounded by lymphatic vessel valve dysfunction in aged mice.

On the other hand, key regulators that regulate cell polarity, such as CELSR1, PKD1, and FAT atypical cadherin 4 (FAT4) are essential for lymphatic vessel valve development^[92–94]^. Mutations in *FAT4* have been associated with Hennekam syndrome, a genetically diverse condition characterized by lymphedema, lymphatic vessel dilatation, and mental retardation, emphasizing the critical role of FAT4 in the lymphovascular system^[95]^. Meanwhile, *FAT4* is a target gene of *GATA2*, a critical transcriptional regulator in the development of lymphatic vessel valves^[91]^.

#### Mechanical signals

Lymphatic vessels also play an essential role in regulating lymphatic flow by sensing mechanical signals. Additionally, the generation of fluid shear induces the production of nitric oxide (NO), leading to local relaxation of lymphatic vessels^[96]^. The increased lymphatic flow influences the expression of chemokines and adhesion molecules in lymphatic endothelial cells, facilitating the transport of immune cells and enabling a swift response to immune challenges. These discoveries underscore the significance of lymphatic flow as a key regulator of lymphatic endothelial cell function^[97]^.

One study has revealed that the expansion of lymphatic vessels during the development of mouse embryos is correlated with ISF pressure and the elongation peak of lymphatic endothelial cells, and that the reduced ISF shortens lymphatic endothelial cell length, which, in turn, diminishes tyrosine phosphorylation of VEGFR3 and inhibits lymphatic endothelial cell proliferation^[98]^. This novel mechanism of VEGFR3 activation through mechanotransduction is crucial for normal development and fluid homeostasis in the mammalian embryo. In addition, the formation of valves was predominantly observed at the branching points of lymphatic vessels, suggesting that different fluid flow patterns exposed to endothelial cells may serve as a mechanical stimulus for the initiation of valve development^[99]^. It has been shown that human lymphatic endothelial cells exposed to an oscillatory but non-laminar shear environment upregulate FOXC2 levels and the activation of calcium-regulated phosphatase/nuclear factor of activated T cells (NFAT) signaling pathway, which are all important for lymphatic vessel valve development^[36]^.

## Targeted interventions

### Drug treatment

AQP4 plays a crucial role in brain water balance and the development of edema^[100–101]^. Using an AQP4 agonist (cyanamide, CYA) and an AQP4 inhibitor (TGN-020), studies have suggested that minocycline treatment for traumatic brain injury preserves blood-brain barrier integrity by decreasing the AQP4 level and restoring the localization of the astrocyte AQP4, which reveals the importance of AQP4 in the amelioration of brain injury by minocycline^[102]^. In a preclinical study, a novel AQP4 agonist (TGN-073) was employed, which demonstrated an increased uptake of intravenous ^17^O-labeled water into the brain, particularly in the cortical PVS through MRI^[103]^. These findings provide a strong evidence for the AQP4-facilitated ISF circulation for clearance effect.

Moreover, AQP4 modulators have emerged as potential drug targets for neurological disorders^[104]^. However, clinical development of AQP4 inhibitors faces challenges, including a poor druggability of AQP, a large number of homologous AQP isoforms with wide distributions and functional similarities, technical difficulties in water transport assays, the prediction of potential adverse side effects, and the need for high blood-brain barrier penetration. To date, despite considerable efforts, no breakthrough has been achieved with the validated small-molecule AQP4 inhibitors. Nevertheless, there is still an AQP monoclonal antibody drug (aquaporumab) treatment in a mouse model of neuromyelitis optica^[105]^. A range of treatments to inhibit AQP4 is already available in the clinic, including tetraethylamine^[106]^, acetazolamide and carbonic anhydrase inhibitors^[107]^, bumetanide-derived loop diuretics^[108]^, and some anticonvulsants^[109]^. However, whether these compounds cause off-target AQP4 inhibition when administered at clinically relevant doses remains a topic of debate^[104,110]^.

An increasing number of experimental animal studies have demonstrated that different forms of VEGFC supplements, such as a recombinant VEGFC protein^[75]^, AAV1-CMV-mVEGF-C156S^[34]^, VEGFC peptide^[34]^ or VEGFC mRNA^[111]^, significantly increase meningeal lymphatic vessel coverage and subsequently improve brain lymphatic drainage functions, thus playing important roles in brain tumors^[111]^, traumatic brain injury^[112]^, AD^[42,75]^, and other diseases. These findings suggest that meningeal lymphatics represent promising targets for intervention in various CNS diseases. In addition, Down syndrome critical region 1 (*DSCR1*), a gene associated with Down syndrome, has been reported to be involved in the regulation of meningeal lymphatic vessel function^[113]^. However, there are no clinical drugs currently available to improve meningeal lymphatic vessel function. Considering the meningeal lymphatics are affected by physical stimulation^[98]^, lifestyle modifications may offer a less invasive, safer, and more implementable way to intervene in the meningeal lymphatics.

### Non-pharmacological interventions

A recent study by Professor Jianping Jia's team in 2023^[114]^ assessed the associations between six key lifestyle factors and memory function in a 10-year cohort study, which involved more than 29000 individuals aged 60 and older in China. The study assessed the impact of physical activity, dietary habits, alcohol consumption, smoking, cognitive engagement and social interaction on memory, and demonstrated that adopting a healthy lifestyle could lower the risk of developing dementia. Furthermore, another study also suggested that implementing healthy lifestyle interventions could substantially increase life expectancy among the Chinese population^[115]^.

#### Healthy lifestyle (about diet, exercise, sleep, and environmental enrichment [EE])

An inappropriate dietary structure significantly contributes to conditions such as obesity, metabolic syndrome, and type 2 diabetes. These metabolic disturbances, in turn, increase the risk of AD^[116]^. One animal study reported that a diet with high polyunsaturated fatty acids (PUFAs) caused an increased CSF flow, improving tracer clearance and the impaired AQP4 polarity^[117]^. This suggests a protective role for PUFAs in maintaining normal lymphatic transport function, which is not obvious in the *Aqp4* knockout mice^[117]^.

Alcohol abuse is well-established as a risk factor for cancer and cardiovascular disease^[118]^. In rats, even a single episode of alcohol abuse has been shown to cause brain damage^[119]^. Conversely, some reports have suggested a lower incidence of PD or dementia among light drinkers, compared with non-drinkers^[120–121]^. However, the effects of alcohol on the glymphatic system also show a J-shaped dose-response curve, with low levels of alcohol appearing to enhance the rapid clearance of CSF solutes, while high levels of alcohol inhibit glymphatic system functions. Chronic moderate alcohol consumption leads to a significant activation of astrocytes and an extensive loss of perivascular AQP4 polarization, resulting in irreversible glymphatic transport impairment^[122]^. Additionally, in a study involving college students aged 18 to 21 years, alcohol abuse was found to alter sleep patterns, including an increasing slow-wave sleep and a decreasing rapid eye movement sleep during the first half of the sleep period but an increasing sleep disruption in the second half of the sleep period^[123]^. This suggests that alcohol may also modulate glymphatic system functions by disrupting sleep patterns.

Exercise as a healthy lifestyle has been reported to reduce cognitive impairment in patients with dementia^[124]^. In rodent studies, voluntary exercise increased CSF flow in the brain during the wakefulness of young mice^[125]^ and attenuated amyloid plaque deposition, improved AQP4 polarity distributions and spatial cognitive memory performance of the aged mice^[126]^. Furthermore, the *Aqp4* knockout APP/PS1 mice had more severe deficits in glymphatic transport functions, Aβ plaque deposition, and cognitive impairment, which were not alleviated after the exercise intervention^[127]^. These findings suggest that the AQP4-dependent glymphatic system is essential for the neuroprotective effects of exercise during the AD process. However, some studies showed that regular wheel running did not alter vascular amyloid burden in the Tg-SwDI mice^[128]^.

Previous studies have revealed that even a single night of sleep deprivation in healthy individuals leads to an increase in the Aβ load in the hippocampus^[129]^. Similarly, the expression levels of Aβ_42_^[130]^, TAU^[131]^ and α-Syn^[131]^ are significantly increased in the CSF after acute sleep deprivation. Furthermore, it was observed a higher Aβ_42_ level in the CSF of patients with chronic insomnia than those who sleep normally^[132]^. These findings have also been replicated in animal studies^[133–135]^. Notably, in humans, single nucleotide polymorphisms in *AQP4* have been linked to the self-reported declines in sleep quality and an increased Aβ accumulation, suggesting that genetic variations in *AQP4* not only affect Aβ accumulation but also influence sleep quality^[136]^.

Professor Maiken Nedergaard *et al* demonstrated in 2020^[60]^ that CSF distribution was governed by circadian rhythms and that AQP4 supported such rhythms. Light and dark cycles drive circadian rhythms; therefore, the changes in light exposure may affect glymphatic transport functions. One study reported that red light exerted a strong sleep-inducing effect, when the intensity was > 20 lux and influenced sleep architecture in terms of sleep duration and number, stage transitions, and electroencephalogram waveforms; subsequently, ≤ 10 lux of red light did not affect sleep-wake behavior by reducing the light intensity^[137]^. These findings highlight the importance of limiting the intensity of red light (≤ 10 lux) to avoid the effects of light on sleep and circadian rhythms in nocturnal behavioral experiments. Therefore, phototherapy has also been applied to treat excessive daytime sleepiness in PD patients^[138]^.

Enriched environments have significant effects on brain plasticity and neuropathology, which provides novel avenues for gene-environment interactions in neurodegenerative diseases^[139–140]^. In addition, mouse hippocampal CA1 bilaterally injected with short hairpin RNA (shRNA) targeting acutely silenced *Aqp4*, following exposure to EE, which counteracted EE-induced increase of hippocampal volume and astrocyte branching and density^[141]^. Moreover, exercise is a vital component of EE, and the effects of motor activity on the brain are enhanced by voluntary exploratory movements or forced running. Thus, EE may also regulate the brain lymphatic system through motor stimulation. Apart from this, EE also enhances sensory stimulation, including an increased somatosensory, visual, and olfactory input, and an increased cognitive stimulus, such as spatial maps and novel objection recognition, making multifaceted interventions^[140]^.

#### Physical therapy

In addition, some emerging physical therapies, such as repetitive transcranial magnetic stimulation (rTMS) and transcranial photobiomodulation (tPBM), have also been reported to alleviate the disease process by targeting interventions in the brain lymphatic system.

rTMS is a painless, noninvasive method for the treatment of CNS disorders. Continuous theta-burst stimulation (CTBS) is a variable mode of rTMS. One study showed that the APP/PS1 mice with CTBS treatment significantly improved AQP4 polarization, lymphatic fluid transport and CSF-ISF exchange, which in turn reduced Aβ deposition and enhanced spatial learning and memory^[142]^. Moreover, another study revealed that CTBS also significantly improved the impaired AQP4 polarity and glymphatic drainage in the sleep-deprived mice and attenuated anxiety-like behaviors^[143]^. Furthermore, it was reported that acute CTBS upregulated meningeal VEGFC expression and increased meningeal lymphatic vessel diameter, while sunitinib treatment was used to temporarily suppress VEGFR levels and block the VEGFC-VEGFR3 signaling pathway, which counteracts the lymphatic vessel diameter expanding effect of CTBS; moreover, long-term CTBS was found to change lymphatic vessel coverage, length and branching^[144]^. Therefore, the different modes of sequential CTBS intervention deserve further exploration. However, whether CTBS contributes to meningeal lymphatic drainage has not been clarified.

Lin *et al*^[145]^ also showed that a high-frequency of rTMS treatment significantly reduced Aβ deposition and glial cell activation in the brain, improving the functions of the glymphatic system and meningeal lymphatic vessels of the 4 to 5-month-old 5 × FAD mice. Accordingly, rTMS treatment effectively blocked the decline in working memory in the 5 × FAD mice during experimental tests of a novel object and new location recognition. In conclusion, these data provide a theoretical foundation and clinical application for rTMS modulating brain lymphatic drainage and thus attenuating early AD symptoms.

tPBM is a therapy that involves the irradiation of red laser light (in the range of 5–600 nm) or near-infrared light (in the range of 700–760 nm) into the head through the intact scalp and skull. The light penetrates the brain and is absorbed by specific chromophores, stimulating the production of adenosine triphosphate and NO, enhancing the metabolism of neurons in brain tissue as well as anti-apoptotic and antioxidant capacity^[146]^. It was reported that tPBM treatment (1267 nm, 32 J/cm^2^) attenuated Aβ plaque deposition and impaired learning memory in the AD model mice, compared with the control-treated mice; moreover, tPBM treatment was further demonstrated to improve glymphatic functions of Aβ clearance by the application of optical coherence tomography in mice^[147]^. Another study reported that the 630 nm red light treatment also attenuated Aβ deposition in the APP/PS1 mice, restored impaired ISF flow, and further improved the learning memory ability of the APP/PS1 mice in the Morris water maze; besides, tPBM treatment (10 J/cm^2^) significantly enhanced the drainage function of meningeal lymphatics and promoted the excretion of exogenous gold nanorods injected in the cortical region to the deep cervical lymph nodes^[148]^. These results suggest that tPBM may be a promising therapeutic target for the prevention or delay of AD.

Moreover, tPBM, as a light energy-based therapy, is influenced by circadian rhythms. Therefore, when to apply red or near-infrared light intervention has the greatest benefit for glymphatic transport and meningeal lymphatics deserves further investigation.

## Summary and prospects

Aging and age-related neurodegenerative diseases are common challenges throughout the world. The accumulation, misfolding, or mislocalization of toxic proteins that lead to neuronal loss are key pathological hallmarks of neurodegenerative diseases. In addition, the brain lymphatic system mediates the clearance of metabolic waste from the brain parenchyma, which provides new perspectives to investigate neurodegenerative diseases. Therefore, it is crucial to understand whether the fundamental anatomical structures and physiological functions of the brain lymphatic system are conserved among species. Most of the present studies have been carried out in rodents, but some human findings support the existence of a glymphatic system and meningeal lymphatic pathway^[13,149–150]^. Measuring apparent diffusion coefficients using diffusion tensor imaging may be used to investigate the mechanisms of fluid transport in the brain^[150]^. Previous MRI data demonstrated that CSF contrast entered the brain parenchyma and subsequently cleared, whereas patients with dementia showed a delayed clearance of the contrast^[13]^. Therefore, measuring CSF clearance may have predictive value in the diagnosis of clinical dementia.

So far, there is no available treatment for neurodegenerative diseases. Dissecting the influences of healthy aging and disease risk factors assists us in assessing which lifestyles may constitute a major risk for neurodegenerative diseases. Animal studies have also shown that environment, diet, and sleep may modulate brain lymphatic drainage. Epidemiological studies have shown that lifestyle-related factors may delay the risk of developing neurodegenerative diseases^[114]^. Thus, having a healthy lifestyle will benefit us for life.

**Figure 3:**
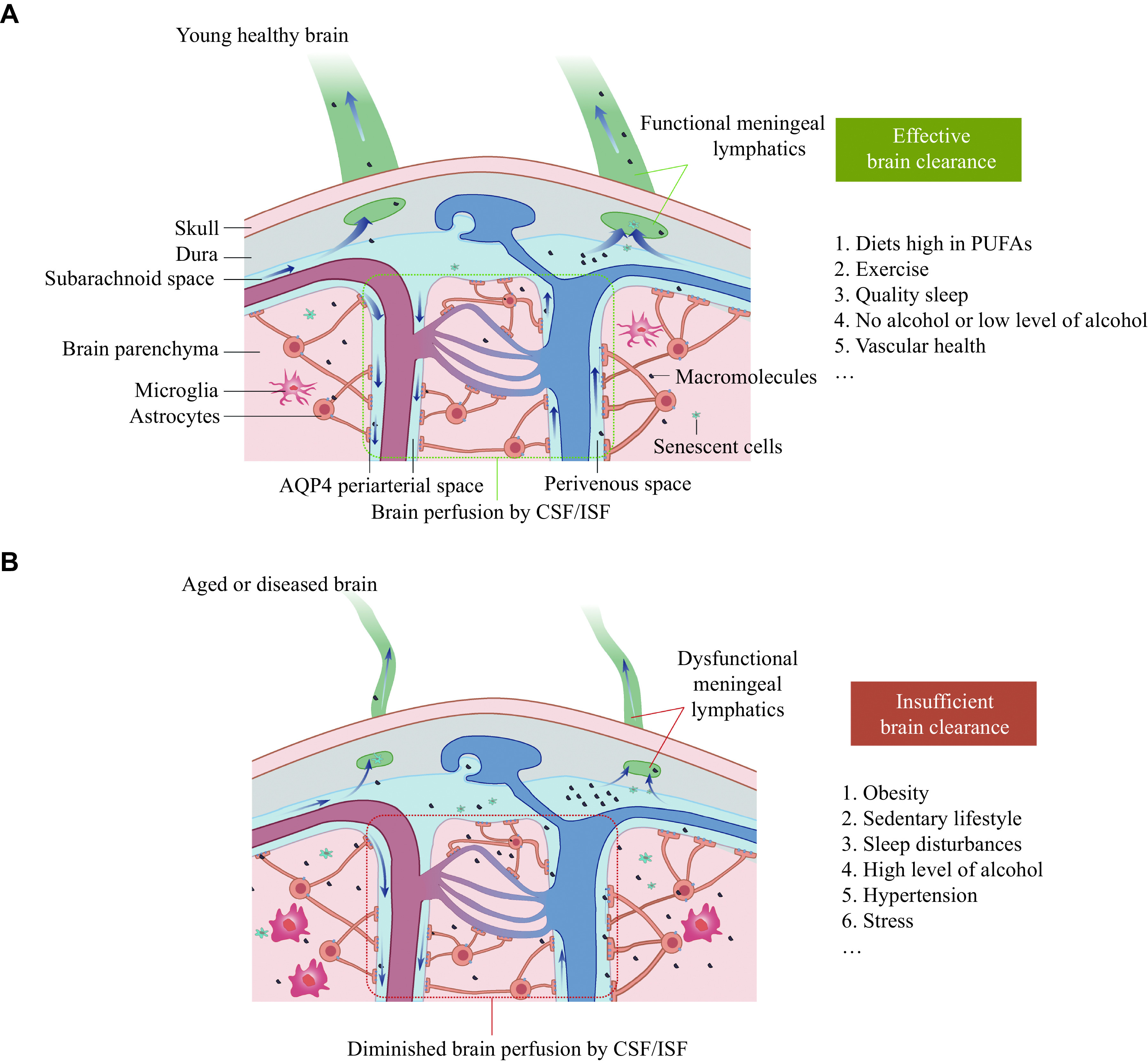
Targeted interventions for the impaired function of the glymphatic system and meningeal lymphatic vessels in aging and neurodegenerative diseases.

The glymphatic system and meningeal lymphatic vessels play critical roles in the drainage of metabolites to maintain brain homeostasis. These drainage pathways are functionally impaired in aging and neurodegenerative diseases, which further exacerbate disease progression. In the present review, we have elucidated potential mechanisms of the glymphatic system and meningeal lymphatic vessels in regulating lymphatic drainage, offering prospects for novel interventions that may serve as targets for treating neurological diseases by modulating brain lymphatic drainage through various means (***Fig. 3***).
